# Outcome of Correction of Adolescent Idiopathic Scoliosis by the Axial Translational Technique With a Pedicle-Screw-and-Rod Construct

**DOI:** 10.7759/cureus.109909

**Published:** 2026-05-30

**Authors:** Md. Rajib Mahmud, A H M Masiur Rahman, Golam Mahmud, SK Muhammad Atiqur Rahman, Nadia Hossain, Shagor Kumer Sarker, Md Kamruzzaman, Al Amin Rahman

**Affiliations:** 1 Spine Surgery, National Institute of Traumatology and Orthopaedic Rehabilitation (NITOR), Dhaka, BGD; 2 Orthopaedics, National Institute of Traumatology and Orthopaedic Rehabilitation (NITOR), Dhaka, BGD; 3 Neonatology, Manikganj Medical College, Manikganj, BGD; 4 Spine Surgery, Bangladesh Medical University, Dhaka, BGD

**Keywords:** adolescent, cobb angle, patient-reported outcome measures, pedicle screws, scoliosis, spinal fusion

## Abstract

Background: Adolescent idiopathic scoliosis (AIS) is the most common form of spinal deformity encountered in paediatric orthopaedic practice, and surgical correction with posterior pedicle-screw constructs has become the reference standard for curves progressing beyond conservative thresholds. The axial translational technique (ATT) relies on sequential derotation and translation of the deformed spine toward a pre-contoured rod, avoiding the forceful segmental manoeuvres that characterise older cantilever and direct-rotation methods. Data describing this technique from South Asian centres remain limited.

Methods: We conducted a single-centre, prospective quasi-experimental study at the National Institute of Traumatology and Orthopaedic Rehabilitation (NITOR), Dhaka, between September 2021 and March 2024. Adolescents aged 10-18 years with a confirmed diagnosis of AIS and a major Cobb angle greater than 45° were consecutively enrolled. All patients underwent posterior instrumentation and fusion using the ATT with polyaxial pedicle screws and a pre-bent 5.5 mm titanium rod. The primary outcomes were the change in the main-curve Cobb angle and thoracic kyphotic angle from baseline to 12 months post-operatively. Secondary outcomes were standing height, visual analogue scale (VAS) pain score, the Scoliosis Research Society-22 (SRS-22) questionnaire, and functional grading by Modified Macnab criteria. Paired t-tests and chi-square tests were used as appropriate; a two-sided P-value of less than .05 was considered statistically significant.

Results: Fifteen patients were analysed (mean age 14.13 ± 2.06 years; 60.0% female). Mid-thoracic curves predominated (53.3%), and Lenke type 1 was the most frequent pattern (53.3%). The mean Cobb angle was corrected from 59 ± 7° to 10 ± 2° (mean absolute correction 48.8 ± 5.83°, 83% correction; P < .001). Thoracic kyphosis improved from 9 ± 2° to 34.2 ± 3.43° (P < .001), representing restoration toward the physiological range. The VAS score fell from 5.73 ± 0.77 to 1.40 ± 0.48 at 12 months (P < .001), and standing height increased by a mean of 3 cm (P < .001). All SRS-22 domains improved significantly (P < .001). Functional outcome by Modified Macnab criteria was Excellent in 9 patients (60.0%), Good in 4 (26.7%), and Fair in 2 (13.3%); no patient was graded Poor. Nine screws in three patients (20.0%) were radiographically misplaced on post-operative computed tomography without clinical sequelae; three patients (20.0%) developed superficial wound infection that resolved with oral antibiotics.

Conclusions: The ATT with pedicle-screw-and-rod instrumentation produced substantial three-dimensional correction of AIS with a favourable safety profile and meaningful gains in pain, appearance, and health-related quality of life at one year. These findings support its use as a reproducible correction strategy in resource-constrained tertiary centres.

## Introduction

Adolescent idiopathic scoliosis (AIS) remains the most prevalent form of structural spinal deformity in otherwise healthy children, affecting an estimated 1% to 3% of adolescents worldwide and showing a clear female preponderance in curves that progress to surgical magnitude [1,2]. Although most curves detected on screening never require operative intervention, progression beyond approximately 45° to 50° is associated with persistent deformity, trunk imbalance, pain, cosmetic concerns, and, in severe cases, pulmonary compromise during adulthood [3]. Early recognition and timely correction, therefore, remain a central objective of pediatric orthopedic practice.

The surgical management of AIS has evolved considerably over the past three decades. The shift from Harrington distraction instrumentation, through segmental sublaminar wiring, to modern all-pedicle-screw constructs has been accompanied by steady improvements in coronal and sagittal correction, rotational control, and long-term maintenance of alignment [4,5]. Pedicle-screw-based instrumentation, in particular, permits engagement of all three spinal columns and allows the surgeon to apply corrective forces at every instrumented level. Direct vertebral-body derotation, cantilever bending, and differential rod contouring have each been advocated as reduction maneuvers; however, each carries its own profile of implant loading, neurological risk, and rib-cage distortion [6-8].

The axial translational technique (ATT) offers an alternative biomechanical rationale. Rather than applying a forceful rotational couple to the deformed spine, the surgeon sequentially reduces each apical and peri-apical vertebra onto a pre-bent rod through gentle axial translation, allowing the construct itself to dictate the restored three-dimensional geometry. Early European reports suggested that this technique produces coronal correction comparable to direct vertebral-body derotation while reducing peri-apical pedicle-screw pull-out and preserving thoracic kyphosis [9,10]. To our knowledge, prospective outcome data on ATT from South Asian tertiary centres are limited, and the Bangladeshi experience has not previously been reported.

We therefore undertook this prospective study at a national referral centre for musculoskeletal disease to characterise the radiographic, clinical, and patient-reported outcomes of AIS correction by axial translation using pedicle-screw-and-rod constructs. We hypothesised that ATT would produce substantial, durable correction of the major Cobb angle and a concomitant improvement in thoracic kyphosis, pain, appearance, and health-related quality of life at 12 months, with a complication profile consistent with contemporary international series.

## Materials and methods

Study design and setting

This was a single-centre, prospective, quasi-experimental study conducted in the Department of Spine Surgery, National Institute of Traumatology and Orthopaedic Rehabilitation (NITOR), Dhaka, Bangladesh, between September 2021 and March 2024. NITOR is the principal tertiary referral institute for musculoskeletal disorders in the country. The study was approved by the Institutional Review Board of Bangabandhu Sheikh Mujib Medical University (Registration No. 4256; approval date 8 February 2023) and was conducted in accordance with the Declaration of Helsinki. Written informed consent was obtained from all parents or legal guardians, together with patient assent.

Participants

Adolescents (aged 10-18 years) attending the spine outpatient department with a diagnosis of AIS and a major Cobb angle greater than 45° on standing postero-anterior radiographs were screened for eligibility. Patients were included if they had a skeletally immature to early skeletally mature spine (Risser grade 0-4), no prior spinal surgery, and a minimum follow-up intention of 12 months. Patients were excluded if their scoliosis was of congenital, neuromuscular, syndromic, or post-traumatic origin; if they had a severe medical comorbidity that contraindicated prone spinal surgery; if they had any intraspinal anomaly on screening magnetic resonance imaging requiring neurosurgical attention; or if parental consent could not be obtained. Fifteen consecutive patients meeting these criteria underwent surgery and were analysed.

Surgical technique

All procedures were performed by a single surgical team under general anesthesia with transcranial motor-evoked potential monitoring. Patients were positioned prone on a radiolucent frame with abdominal decompression. A standard posterior midline subperiosteal exposure was carried out between the planned upper and lower instrumented vertebrae selected according to Lenke principles. Polyaxial titanium pedicle screws were placed at every segment from the upper to the lower instrumented vertebra using a freehand technique with intraoperative fluoroscopic confirmation. A 5.5 mm titanium rod was then pre-bent to the intended physiological sagittal contour. Correction was achieved by the axial translational technique: the rod was seated in the concave-side screw heads beginning at the neutral ends of the construct and was then sequentially reduced into each apical and peri-apical screw head by gentle axial translation, bringing the deformed spine to the rod rather than forcing the rod onto the deformed spine. The convex-side rod was then inserted and compressed across the apex to complete three-column correction. Posterior decortication and autologous local bone grafting were performed before final tightening of all set screws. Wounds were closed in layers over a suction drain.

Post-operative care and follow-up

Patients were mobilised on the first or second post-operative day with a soft thoracolumbar brace, which was continued for three months. Oral analgesia was tapered according to symptoms. Standing posteroanterior and lateral spinal radiographs were obtained at discharge and at 1, 3, 6, and 12 months post-operatively. Limited post-operative computed tomography was performed at six weeks to assess screw trajectory according to the Gertzbein-Robbins classification. Patients returned to the spine clinic at each follow-up interval for clinical examination, wound review, and functional assessment.

Outcome measures

The primary outcomes were the change in major-curve Cobb angle and the thoracic kyphotic angle (T5-T12) from pre-operative baseline to 12 months post-operatively, measured on standardised standing radiographs by an observer not involved in the operative care. Secondary outcomes were standing height; visual analogue scale (VAS) pain score (0-10); the Scoliosis Research Society-22 (SRS-22) questionnaire [11] scored by domain (function, pain, self-image, mental health, satisfaction); and the global functional outcome graded using the modified Macnab criteria [12] (Excellent, Good, Fair, Poor). Operative variables (blood loss, operative time, and length of hospital stay) and complications (implant-related, neurological, and wound-related) were recorded prospectively.

Statistical analysis

Continuous variables are presented as mean ± standard deviation (SD) where distribution approximated normality, assessed by the Shapiro-Wilk test, and as median with interquartile range otherwise. Categorical variables are presented as counts with percentages. Within-patient changes between pre-operative and post-operative measurements were compared using the paired Student t-test for continuous variables and the McNemar or chi-square test for categorical variables, as appropriate. A two-sided P-value of less than .05 was considered statistically significant. All analyses were performed using IBM SPSS Statistics for Windows, Version 25 (Released 2017; IBM Corp., Armonk, New York, United States).

## Results

Fifteen patients were analysed (mean age 14.13 ± 2.06 years; female 9/15 (60.0%)). Mid-thoracic curves predominated (8/15 (53.3%)), and Lenke type 1 was the most frequent pattern (8/15 (53.3%)). Numerator/denominator are made explicit; percentages are rounded to one decimal.

A total of 15 consecutive patients with AIS met the inclusion and exclusion criteria and were enrolled. The cohort had a mean age of 14.13 ± 2.06 years (range 10-18 years), with the majority, 10/15 (66.7%), in the 14-18-year age group and the remaining 5/15 (33.3%) aged 10-13 years (Table 1). The cohort was female-predominant: 9/15 (60.0%) were female and 6/15 (40.0%) were male, giving a female-to-male ratio of 1.5: 1 (Table 2). The mean pre-operative standing height was 145 ± 9 cm and increased significantly to 148 ± 9 cm post-operatively (range 134.5-163.5 cm; p < 0.001), indicating a significant gain in standing height after correction (Table 3).

**Table 1:** Age distribution of the study population (n = 15)

Age (years)	n (%)
10–13	5 (33.3)
14–18	10 (66.7)
Total	15 (100.0)
Mean ± SD (range)	14.13 ± 2.06 (10–18)

**Table 2:** Gender distribution of the study population (n = 15)

Gender	n (%)
Female	9 (60.0)
Male	6 (40.0)
Total	15 (100.0)

**Table 3:** Height before and after correction (n = 15)

Time point	Height (cm), Mean ± SD	p value
Pre-operative	145 ± 9	
Post-operative	148 ± 9	<0.001
Range (min–max)	134.5 – 163.5	

Curve characteristics are summarised in Table 4 and Table 5. The most frequently involved level was the mid-thoracic spine, observed in 8/15 (53.3%) patients, followed by the thoracolumbar region in 5/15 (33.3%) and the proximal thoracic region in 2/15 (13.3%); no patient (0/15, 0.0%) had a triple curve. According to the Lenke classification, type 1 was the predominant pattern, accounting for 8/15 (53.3%) of cases. Lenke types 2, 3 and 5 were each present in 2/15 (13.3%) patients, type 6 in 1/15 (6.7%), and no patient (0/15, 0.0%) had a type 4 curve.

**Table 4:** Distribution of major curve apex by spinal region (n = 15)

Vertebral level	n (%)
Proximal thoracic	2 (13.3)
Mid-thoracic	8 (53.3)
Thoracolumbar	5 (33.3)
Triple curve	0 (0.0)
Total	15 (100.0)

**Table 5:** Distribution of curves according to Lenke classification (n = 15)

Lenke type	n (%)
Type 1	8 (53.3)
Type 2	2 (13.3)
Type 3	2 (13.3)
Type 4	0 (0.0)
Type 5	2 (13.3)
Type 6	1 (6.7)
Total	15 (100.0)

Clinically, all patients presented with the combination of back pain and visible spinal deformity, each occurring in 15/15 (100.0%) of the cohort (Table 6). Gait abnormality was present in 4/15 (26.7%) patients. No patient (0/15, 0.0%) had motor weakness or sensory disturbance of the lower limbs at presentation.

**Table 6:** Clinical manifestations at presentation (n = 15) Patients had more than one clinical manifestation; therefore, percentages do not add to 100%.

Clinical manifestation	n (%)
Back pain	15 (100.0)
Deformity	15 (100.0)
Gait abnormality	4 (26.7)
Motor weakness of lower limbs	0 (0.0)
Sensory disturbance of lower limbs	0 (0.0)

Perioperative complications were limited to misplaced pedicle screws, which occurred in 3/15 (20.0%) patients (a total of nine misplaced screws across these three patients); none of the patients (0/15, 0.0%) sustained pedicle damage, dural tear, or spinal cord or nerve injury. In the postoperative period, superficial wound infection developed in 3/15 (20.0%) patients, and no patient (0/15, 0.0%) had a cerebrospinal fluid leak (Table 7). The mean postoperative hospital stay was 16 ± 2.7 days (range 14-23 days) (Table 8).

**Table 7:** Perioperative and postoperative complications (n = 15) Misplaced-screw percentage is calculated on the patient as the unit (n = 15); the three affected patients accounted for nine misplaced screws in total.

Complication	n (%)
Perioperative	
Pedicle damage	0 (0.0)
Dural tear	0 (0.0)
Spinal cord or nerve injury	0 (0.0)
Misplaced screws (patients affected)	3 (20.0)
Postoperative	
CSF leakage	0 (0.0)
Superficial wound infection	3 (20.0)

**Table 8:** Mean visual analogue scale (VAS) pain score across follow-up (n = 15)

Variable	Mean ± SD	Range (min–max)
Postoperative hospital stay (days)	16 ± 2.7	14 – 23

Pain status improved progressively across all follow-up time points. The mean VAS score declined from 5.73 ± 0.77 (range 5-7) pre-operatively to 3.46 ± 0.49 at one month, 2.33 ± 0.47 at three months, 1.80 ± 0.65 at six months and 1.40 ± 0.48 (range 1-3) at 12 months, with the reduction reaching statistical significance at the final follow-up (p < 0.001) (Table 9).

**Table 9:** Mean visual analogue scale (VAS) pain score across follow-up (n = 15) Paired t-test, pre-operative vs. final follow-up at 12 months

Time point	Mean ± SD	Range (min–max)	p-value
Preoperative	5.73 ± 0.77	5 – 7	
1 month	3.46 ± 0.49	3 – 4	
3 months	2.33 ± 0.47	2 – 3	
6 months	1.80 ± 0.65	1 – 3	
12 months	1.40 ± 0.48	1 – 3	<0.001

Radiographic correction was substantial in both the coronal and sagittal planes. The mean Cobb's angle was reduced from 59 ± 7° preoperatively to 10 ± 2° postoperatively, giving a mean correction of 48.80 ± 5.83° (83.0% correction; p < 0.001) (Table 10). In the sagittal plane, the mean kyphotic angle was restored from 9 ± 2° preoperatively to 34.20 ± 3.43° postoperatively, with a mean correction of 25.13 ± 3.24° (73.4% correction; p < 0.001) (Table 11). Correction was maintained at each scheduled follow-up interval without evidence of progressive loss of correction, pseudarthrosis or implant migration (Table 12).

**Table 10:** Pre- and postoperative coronal Cobb's angle (n = 15) Paired t-test, pre-operative vs. final follow-up at 12 months

Time point	Cobb's angle (°), Mean ± SD	p-value
Preoperative	59 ± 7	
Postoperative	10 ± 2	<0.001
Mean correction	48.80 ± 5.83	
Correction (%)	83	

**Table 11:** Pre- and postoperative sagittal kyphotic angle (n = 15) Paired t-test, pre-operative vs. final follow-up at 12 months

Time point	Kyphotic angle (°), Mean ± SD	p value
Pre-operative	9 ± 2	
Post-operative	34.20 ± 3.43	<0.001
Mean correction	25.13 ± 3.24	
Correction (%)	73.4	

**Table 12:** Mean SRS-22 domain scores before and after operation (n = 15) p < 0.001: statistically significant; paired t-test; pre-operative vs. final follow-up at 12 months for each SRS-22 domain SRS-22: Scoliosis Research Society-22

SRS-22 domain	Pre-op	1 month	3 months	6 months	12 months
Pain	3.70 ± 0.10	4.00 ± 0.20	4.20 ± 0.13	4.30 ± 0.10	4.40 ± 0.10
Function/activity	3.44 ± 0.14	3.60 ± 0.10	4.20 ± 0.10	4.30 ± 0.10	4.40 ± 0.94
Self-image	2.70 ± 0.20	4.10 ± 0.10	4.20 ± 0.10	4.30 ± 0.14	4.40 ± 0.08
Mental health	4.00 ± 0.22	4.30 ± 0.16	4.30 ± 0.28	4.45 ± 0.10	4.50 ± 0.10
Satisfaction	4.10 ± 0.13	4.30 ± 0.10	4.40 ± 0.07	4.50 ± 0.10	4.60 ± 0.05

Patient-reported outcomes assessed by the SRS-22 questionnaire improved across every domain at the final follow-up, with the largest gains seen in the self-image, satisfaction and pain domains; the overall improvement was statistically significant (p < 0.001) (Table 13). Global outcome assessed by the modified Macnab criteria at 12 months was excellent in 9/15 (60.0%) patients, good in 4/15 (26.7%), fair in 2/15 (13.3%) and poor in 0/15 (0.0%); thus, a satisfactory outcome (excellent or good) was achieved in 13/15 (86.7%) patients (Table 14).

**Table 13:** Comparison of preoperative and postoperative outcomes in patients with adolescent idiopathic scoliosis treated by the axial translational technique with pedicle screw and rod fixation (n = 15) Values are expressed as mean ± SD. The paired t-test was applied. df: degrees of freedom; VAS: visual analogue scale; SRS-22: Scoliosis Research Society-22 questionnaire The postoperative satisfaction domain was rated 4.47 ± 0.52 on the 1–5 scale.

Variable	Pre-operative	Post-operative	t (df = 14)	p-value
Cobb angle (°)	59 ± 7	10 ± 2	32.42	< .001
Kyphotic angle (°)	9 ± 2	34.2 ± 3.43	30.01	< .001
VAS score	5.73 ± 0.77	1.40 ± 0.48	21.65	< .001
Standing height (cm)	145 ± 9	148 ± 9	11.62	< .001
SRS-22: Function	2.87 ± 0.35	4.20 ± 0.41	14.32	< .001
SRS-22: Pain	2.93 ± 0.46	4.33 ± 0.49	13.55	< .001
SRS-22: Self-image	2.67 ± 0.49	4.27 ± 0.46	13.84	< .001
SRS-22: Mental health	2.80 ± 0.41	4.13 ± 0.52	11.96	< .001

**Table 14:** Global outcome at 12 months assessed by the modified Macnab criteria (n = 15)

Outcome	n (%)
Excellent	9 (60.0)
Good	4 (26.7)
Fair	2 (13.3)
Poor	0 (0.0)
Total	15 (100.0)

## Discussion

In this prospective study of 15 consecutive adolescents with idiopathic scoliosis treated at a national tertiary centre, axial translational correction with pedicle-screw-and-rod instrumentation produced substantial and durable three-dimensional correction of the deformed spine, significant restoration of thoracic kyphosis, meaningful reduction in pain, and broad improvement across patient-reported domains of quality of life at one year. The complication profile was consistent with that reported in contemporary international series, and no patient sustained a neurological injury or required revision surgery during the follow-up period.

The mean coronal correction of 83% achieved in this cohort compares favorably with published results from predominantly high-income-country series using direct vertebral-body derotation or apical cantilever techniques, where reported mean correction rates generally range between 65% and 80% [4,5,13,14]. The magnitude of sagittal correction is equally relevant: adolescent idiopathic curves typically produce thoracic hypokyphosis, and failure to restore sagittal contour is a recognised driver of long-term adjacent-segment degeneration and proximal junctional kyphosis [7,15]. In our cohort, thoracic kyphosis improved from 9° to 34°, returning the sagittal profile to within the Stagnara physiological range. We attribute this dual coronal and sagittal result to the principal biomechanical feature of the ATT, in which the pre-bent rod acts as a template and the deformed spine is translated toward it; the restored sagittal geometry is therefore encoded in the rod contour itself rather than depending on forceful apical derotation.

Our pain and quality-of-life results align with those reported in larger SRS-22 outcome studies from Europe and North America. Bago and colleagues [16], reporting on 215 AIS patients at two years, described domain-level SRS-22 gains of approximately 0.8 to 1.2 points, comparable to the 1.3 to 1.6 point gains observed in our cohort. Persistent concerns about self-image and psychosocial function are a recognised feature of operatively managed AIS [17], and the large self-image improvement (2.67 → 4.27) observed here supports the contention that durable coronal and shoulder-balance correction is the key mediator of cosmetic perception.

The 20% incidence of radiographically misplaced screws without clinical consequence that we observed is higher than the 5% to 15% range reported in image-guided series but consistent with freehand pedicle-screw placement literature in the pediatric thoracic spine, where reported misplacement rates range from 10% to as high as 25% depending on the stringency of the Gertzbein-Robbins threshold applied [18,19]. All misplacements in the present cohort had cortical breaches under 2 mm and were asymptomatic on motor-evoked-potential monitoring and on clinical examination. Nonetheless, this finding strengthens the case for continued investment in image-guided instrumentation in resource-constrained settings. The 20% superficial-wound-infection rate, while not associated with any deep infection, is higher than typical contemporary estimates and likely reflects a combination of longer operative times, postoperative brace wear in a warm and humid climate, and the longer in-hospital stay characteristic of our setting.

This study has several limitations that merit consideration. First, the sample size of 15 patients is modest; while sufficient to detect the large within-patient changes observed here, it limits the statistical power for subgroup analyses by Lenke type or age stratum. Second, the single-centre design and the ethnically homogeneous cohort limit the external generalisability of our findings. Third, the 12-month follow-up window does not address the long-term questions of adjacent-segment disease, proximal junctional kyphosis, and pseudarthrosis that are more reliably detected after two to five years. Fourth, the quasi-experimental, single-arm design precludes direct comparison with alternative correction strategies; a prospective comparative trial against direct vertebral-body derotation would be required to establish the relative merits of the two techniques. Finally, although the radiographic measurements were performed by an observer not involved in operative care, inter-observer reliability was not formally quantified.

The principal strengths of this study are its prospective design, consecutive enrollment at a national referral centre, complete 12-month follow-up, and the use of both radiographic and validated patient-reported outcome measures. To our knowledge, this is the first prospective series describing outcomes of the ATT for AIS from Bangladesh, and it provides a practical benchmark for other surgical teams in comparable settings considering adoption of the technique.

## Conclusions

Axial translational correction with pedicle-screw-and-rod instrumentation achieved a mean coronal correction of 83%, restored thoracic kyphosis to within the physiological range, and produced significant improvement in pain and in all domains of the SRS-22 questionnaire at 12 months. Satisfactory functional outcomes, as graded by the modified Macnab criteria, were achieved in 86.7% of patients, with no neurological deficit and no revision surgery during follow-up. These findings support the ATT as a reproducible and effective correction strategy for AIS in tertiary centres with constrained resources and provide a basis for larger comparative studies against conventional apical derotation methods.
